# Mass spectrometry‐based tear proteomics for noninvasive biomarker discovery

**DOI:** 10.1002/mas.21691

**Published:** 2021-03-24

**Authors:** Erika Ponzini, Carlo Santambrogio, Antonella De Palma, Pierluigi Mauri, Silvia Tavazzi, Rita Grandori

**Affiliations:** ^1^ Materials Science Department University of Milano‐Bicocca Milan Italy; ^2^ Department of Biotechnology and Biosciences University of Milano‐Bicocca Milan Italy; ^3^ Institute for Biomedical Technologies National Research Council (ITB‐CNR) Segrate (MI) Italy; ^4^ COMiB University of Milano‐Bicocca Milan Italy

**Keywords:** lacrimal film, liquid biopsies, peripheral body fluids, personalized medicine, single‐tear analysis, tear collection and fractionation methods

## Abstract

The lacrimal film has attracted increasing interest in the last decades as a potential source of biomarkers of physiopathological states, due to its accessibility, moderate complexity, and responsiveness to ocular and systemic diseases. High‐performance liquid chromatography‐mass spectrometry (LC‐MS) has led to effective approaches to tear proteomics, despite the intrinsic limitations in sample amounts. This review focuses on the recent progress in strategy and technology, with an emphasis on the potential for personalized medicine. After an introduction on lacrimal‐film composition, examples of applications to biomarker discovery are discussed, comparing approaches based on pooled‐sample and single‐tear analysis. Then, the most critical steps of the experimental pipeline, that is, tear collection, sample fractionation, and LC‐MS implementation, are discussed with reference to proteome‐coverage optimization. Advantages and challenges of the alternative procedures are highlighted. Despite the still limited number of studies, tear quantitative proteomics, including single‐tear investigation, could offer unique contributions to the identification of low‐invasiveness, sustained‐accessibility biomarkers, and to the development of personalized approaches to therapy and diagnosis.

**Abbreviations**
2‐DE: two‐dimensional electrophoresis
AD: Alzheimer's disease
AMD: age‐related macular degeneration
CSF: cerebrospinal fluid
DDA: data‐dependent acquisition
DED: dry eye disease
DIA: data‐independent acquisition
DR: diabetic retinopathy
ELISA: enzyme‐linked immunosorbent assay
EV: extracellular vesicle
FDR: false discovery rate
IGKC: immunoglobulin kappa chain
LC: liquid chromatography
*LCN1*
: lipocalin‐1
Lf: lactoferrin
LTQ: linear trap quadrupole
*LYZ*
: lysozyme C
MALDI: matrix‐assisted laser desorption/ionization
MCT: microcapillary tube
MGD: meibomian gland dysfunction
*MMP1*
: matrix metalloproteinase‐1
MS: mass spectrometry
MuS: multiple sclerosis
OGE: off‐gel electrophoresis
PD: Parkinson's disease
POAG: primary open‐angle glaucoma
PTM: post‐translational modification
SCX: strong cation exchange chromatography
SDS‐PAGE: sodium dodecyl sulfate–polyacrylamide gel electrophoresis
SS: Sjögren's syndrome
STS: Schirmer's test strip
SWATH: sequential window acquisition of all theoretical mass spectra
TAO: thyroid‐associated ophthalmopathy
TOF: time of flight
ZAG: zinc‐α2‐glycoprotein

## 1 TEARS AS AN UNCONVENTIONAL SOURCE OF BIOMARKERS

### 1.1 Introduction

Clinical investigation based on proteomics (clinical proteomics) has made great strides, moving from the discovery of single biomarkers for early disease diagnosis to the comprehensive characterization of protein‐expression profiles, able to identify both the specific disease and its subtyping (Hovestadt et al., 2020; Tyler & Bunyavanich, 2019). This approach also allows for the investigation of therapeutic effects and the characterization of specific signatures useful to predict potential responders to treatments (Mauri et al., 2014).

In fact, diseases can lead to perturbation in different biological pathways and usually affect many processes, whose simultaneous analysis has become possible with the advent of high‐throughput “‐omics” approaches. As established by the US institutions FDA and NIH, there are different classes of biomarkers, according to the context of their use in research and clinical practice, but they are identified by the same basic definition: “a biological marker is a characteristic that is objectively measured and evaluated as an indicator of normal biological processes, pathogenic events or pharmacologic responses to a therapeutic intervention” (Califf, 2018). The recent improvements in mass spectrometry (MS)‐based technologies have made possible to revolutionize the study of biomarkers in health care, extending it to the protein level and linking genotype to phenotype. The unprecedented and deepened detection of differentially expressed proteins, along with their systems biology investigation, offers potential markers of clinical interest and insights on the molecular mechanisms of human diseases. Moreover, a detailed stratification of individual subjects by proteomic mapping, for both diagnosis and treatment prediction, is a crucial step to translate personalized medicine into practice. However, one of the critical aspects of this process is the availability of samples that are simple to collect and representative of the physiopathological state. To date, the main representative samples are tissues where the disease is localized, and molecular signatures are more abundant and specific. Of course, collection of disease‐related human tissues is not always simple or possible (e.g., practical and ethical issues of ante‐mortem brain biopsies restrict the collection of brain specimens to post‐mortem tissues) and researchers have resorted to animal models. Nevertheless, the latter are expensive, may yield results untranslatable to humans, and are highlighting social and ethical dilemmas (Yip et al., 2019). Possible alternative surrogate materials to disease‐related tissues are biofluids, such as plasma, urine (Ferrari et al., 2019), and other less conventional liquid biopsies.

MS‐based proteomics of body fluids has strongly impacted the field of biomarker discovery for diseased states in the last decades (Zhou et al., 2017). Plasma is the fluid most widely employed to this purpose, thanks to the deep characterization of its proteome and relative accessibility (Geyer et al., 2017). In recent years, studies on other body fluids as alternative sources of biomarkers have seen a major impulse. Peripheral fluids such as urine, saliva, sweat, fat aspirate, and tears have elicited a growing interest, thanks to their sustainable accessibility and relatively low complexity (Azkargorta et al., 2017; Beasley‐Green, 2016; Brambilla et al., 2012; Castagnola et al., 2011; Csősz et al., 2017; Gerner et al., 2020; Katsani & Sakellari, 2019). Indeed, these samples are usually simple to collect and contain potential disease‐related signals. Nonetheless, these signatures are diluted, merged with proteins from other tissues/organs and overloaded by large amounts of few proteins, such as albumin and immunoglobulin in plasma and, thus, require to be separated from the “biochemical baseline” of the fluid.

At the same time, contributions from different organs and tissues make these body fluids of interest for either organ‐specific or systemic diseases, since they may reflect the status of the entire body, as well (Zhao et al., 2018). For example, urine, sweat, tears, and saliva have provided a considerable number of putative biomarkers for systemic diseases, such as cancer (Ferrari et al., 2019), Sjögren's syndrome (Aqrawi et al., 2017), Vogt–Koyanagi–Harada disease (Cui et al., 2020). This interest in alternative liquid biopsies arises from the invasiveness of plasma collection methods. This issue represents a major disadvantage also concerning other body fluids, such as amniotic and cerebrospinal fluid (CSF). In the same line, nasal lavages have also been recently considered as an attractive source of peripheral biomarkers (Schoenebeck et al., 2015).

Despite the many interesting features of peripheral body fluids, each of them presents its own specific limitations. Urine displays broad variations in protein concentrations depending on daily water intake (Athanasatou et al., 2019); the salivary proteome is heavily affected by the oral cavity microbiome (Katsani & Sakellari, 2019); sweat analysis has to face peculiar volume‐normalization difficulties (Huestis et al., 2000; Mena‐Bravo & Luque de Castro, 2014); tear proteomics deals with unique reproducibility problems linked to the collection method (Rentka et al., 2017). Strategies can be envisaged to mitigate each of these issues, making these biological fluids attractive reservoirs of potential, complementary biomarkers.

In the trade‐off between the “near the source” and “at the periphery” search for biomarkers, localized biofluids, such as tears, saliva, and nasal lavages, may represent a valuable compromise, offering the advantages of peripheral body fluids for sample collection and the advantages of organ/tissue‐specific biofluids for protein profiling. This review focuses on the recent advances in tear, MS‐based proteomics in the frame of research on low‐invasiveness, human, personalized biomarkers. Besides continuously accessible, tears offer an inexpensive source of biological fluid, minimal storage requirements (frozen samples at −20°C to −80°C can be stored for years without degradation) (de Jager et al., 2009; Hu et al., 2006), high protein concentration (ranging approximately between 4 and 10 μg/μl in basal, open‐eye tears) (Lawrenson, 2018; Phillips & Speedwell, 2018; Sack et al., 2003), and responsiveness to systemic and ocular conditions (Zhou & Beuerman, 2017).

For the same reasons, tear proteomics has received growing attention also in studies on animal models, such as dogs, cows, sheep, and camels (Chen et al., 2011; Shamsi et al., 2011; Winiarczyk et al., 2015). Biomarker discovery by tear proteomics has been reported for a rabbit model of Sjögren's syndrome (Zhou et al., 2013) and canine cancer (de Freitas Campos et al., 2008). Knowledge of the tear film structure has been continuously evolving. Literature reports refer to either a two‐layer or a three‐layer description (Willcox, 2019). In the former, the tear film is composed of an outer lipid layer and an inner aqueous layer containing proteins, metabolites, electrolytes and mucins, transmembrane glycoproteins (Pflugfelder & Stern, 2020). In the latter, mucins constitute a distinct, third gel layer that maintains the hydration and lubrication of the ocular surface and reduces the shear stress and damage of the corneal epithelial cells (Mastropasqua et al., 2019). The composition of these layers is expected to reflect the pathophysiological state of the tissues underneath and the whole body, making it suitable for the evaluation of health and diseased states.

Three main types of tear can be collected, basal, reflex, and psycho‐emotional (Murube, 2009). Each type displays peculiar characteristics. Basal tears, also known as non‐stimulated tears, coat the eye to keep it moist and protected. On the other hand, reflex (or stimulated) tears represent the natural response to irritants, such as wind or dust. Psycho‐emotional tears do not depend on the environment, but on the emotional state of the subject, and are caused by very intense emotions, such as happiness or sadness. Tear composition is also influenced by the possibility of opening or closing the eye: open‐eye tears are different from closed‐eye tears, which are collected after a prolonged closure, typically right after sleeping (Sitaramamma et al., 1998). Protein concentration and collectable sample volume are inversely proportional: reflex tears provide the highest sample volume, whereas closed‐eye tears display the highest protein concentration (Sitaramamma et al., 1998).

The recent progress in MS‐based tear proteomics is discussed here, with a focus on the most critical analytical steps for high‐performance, quantitative analysis. Altogether, the state of the art indicates that the analysis of the human tear proteome offers a promising opportunity in biomarker discovery and personalized medicine for human ocular and systemic diseases.

### 1.2 Tear proteome of healthy human subjects

A crucial starting point for biomarker discovery in tear proteome is the in‐depth investigation of tear proteins in healthy subjects. To the best of these authors’ knowledge, to date only five studies focused on the investigation of the tear proteome of healthy human subjects, with the goal of optimizing the analytical procedure and maximizing proteome coverage (described in Table 1). The authors and co‐workers are currently working on a subsequent paper in this field. The number of protein tear identifications has remarkably increased since the first publication (Li et al., 2005), achieving the highest proteome coverage (1543 proteins) in a paper published in 2012 (Zhou et al., 2012). One of the major challenges in tear proteomics is the broad dynamic range, due to the presence of a few highly expressed proteins (de Souza et al., 2015). Among these, the most abundant one is lactotransferrin, also known as lactoferrin (Lf), which plays a fundamental role in anti‐inflammatory and antimicrobial responses (Flanagan & Willcox, 2009; Pastori et al., 2015, 2019; Ponzini et al., 2020) and has also been recently recognized as an effective inhibitor of SARS‐CoV‐2 infection (Lang et al., 2011; Mirabelli et al., 2020; Zhou et al., 2020). The high concentration of Lf highlights the role of the tear film in maintaining a clean environment for the eye. Other highly concentrated proteins are lipocalin‐1 (*LCN1*), serum albumin, lysozyme C (*LYZ*), and several immunoglobulins. Some of these proteins are secreted in the tear film directly by lacrimal glands (e.g., Lf and *LCN1*) or by lysosomes (e.g., *LYZ*), whereas others are serum proteins (e.g. albumin), which are present in the tear film thanks to blood vessel permeability. Another source of proteins is represented by cells infiltrating the conjunctiva, such as T and B cells, which produce immunoglobulins and cytokines under certain conditions (Offiah & Calder, 2009).

**Table 1:** Comparison of the five human tear proteome studies concerning healthy subjects published since 2005

Study	Sample (*n*)	Collection method	Amount used	Fractioning	No. of fractions	Enzymatic digestion	Online LC column	Gradient length, flow	Spectrometer	MS mode	Data analysis software	No. of protein IDs
Li et al. (2005)	One individual, single take	Open‐eye MCT	1 µl SDS‐PAGE	SDS‐PAGE	10	Trypsin	None	None	Reflex III MALDI‐TOF (Bruker)	Full scan	Mascot	17
			5 µl in solution	None	0	Trypsin	150 µm × 150 mm, ND particle size, ND Å	120–200 min, 1 µl/min	LCQ Deca (Thermo); QSTAR Pulsar QqTOF (ABSciex)	DDA	Mascot	52
de Souza et al. (2006)	One individual, different takes	Closed‐eye MCT	4 µl SDS‐PAGE	SDS‐PAGE	13	Trypsin	ND	ND	LTQ‐FT (Thermo)	DDA	Mascot and MSQuant	320
			1 or 4 µl in solution	None	0	1. Lys‐C	ND	ND	LTQ‐FT, LTQ‐Orbitrap (Thermo)	DDA	Mascot and MSQuant	63
						2. Trypsin						
Zhou et al. (2012)	Pool (4 individuals)	STS	~400 µg TP	Off‐line SCX	6	Trypsin	75 µm × 500 mm, 2 µm particle size, 100 Å	60 min, 300 nl/min	Triple TOF 5600 (ABsciex)	DIA	ProteinPilot	1543
Aass et al. (2015)	Pool (3 individuals)	STS	ND	Off‐line SCX	16	1. Lys‐C	1.0 × 250 mm, 5 µm particle size, 100 Å	180 min, 40 µl/min	LTQ Orbitrap XL (Thermo)	DDA	Proteome Discoverer	1526
						2. Trypsin						
Dor et al. (2019)	Pool (2 or 3 individuals)	STS	60 µg TP	Off‐gel electrophoresis	12	Trypsin	75 µm × 150 mm, 5 µm particle size, 100 Å	85 min, 220 nl/min	LTQ Orbitrap Velos Pro (Thermo)	DDA	Proteome Discoverer	1351

Abbreviations: DDA, data‐dependent acquisition; DIA, data‐independent acquisition; FT, Fourier transform; LC, liquid chromatography; LTQ, linear trap quadrupole; MALDI, matrix‐assisted laser desorption ionization; MS, mass spectrum; MCT, microcapillary tube; ND, not defined; SCX, strong cation exchange chromatography; SDS‐PAGE, sodium dodecyl sulfate–polyacrylamide gel electrophoresis; STS, Schirmer's test strip; TOF, time‐of‐flight; TP, total proteins.

The tear proteome is highly dynamic. For example, variable levels of hormones have been reported to be connected to meibomian and lacrimal gland function (Schirra et al., 2006). Age and sex could represent a major source of variability on tear protein expression. As expected, age has been reported to be directly correlated with many proteins playing important biological functions such as cell death, inflammatory and immune response. In particular, the elder group (60+ years) displayed elevated levels of interleukin 8, 6 and matrix metalloproteinase‐1, when compared with the young (18–40 years) and middle‐aged (41–60 years) groups, whereas the middle‐age group had increased levels of interleukin 7 compared to the young group. On the other hand, sex alone did not affect significantly the protein expression levels in age‐matched groups (Micera et al., 2018).

Inter‐day variability was evaluated on a proteome fraction (peptides and proteins in the 1–20 kDa range), showing no significant differences in tear samples collected from the same individuals in a time window of seven days (González et al., 2012). In this case, neither sex nor age effects were observed. Proteome stability was further investigated concerning cytokine and chemokine levels, which displayed no significant inter‐day differences and, however, notable intra‐day variability as a function of the sample collection time (Benito et al., 2014; Uchino et al., 2006). Indeed, tear cytokine levels were generally higher in the evening than in the midday samples. An opposite trend was observed for matrix metalloproteinase 9, whose concentration was higher after the awakening and negligible during the day (Markoulli et al., 2012). These results suggest that withdrawal time must be controlled to allow for meaningful comparisons.

### 1.3 Biomarker discovery in ophthalmology

Distinct proteomics approaches highlighted differences in the tear proteome profile for several ocular diseases (described in Table 2). Quantitative proteomics can be particularly useful in discriminating among pathologies presenting similar symptoms, such as *meibomian gland dysfunction* (MGD), *dry eye disease* (DED), and *Sjögren's syndrome* (SS). A comparative analysis focused on MGD and DED has revealed differences in the expression levels of 26 proteins (Soria et al., 2013, 2017). A similar analysis was performed on patients affected by DED or SS (Tomosugi et al., 2005). In both comparisons, MS‐based proteomics allowed to differentiate not only between control and diseased state, but also between the pathologies. This result is particularly relevant because DED can occur in association with other pathologies, including MGD and SS (Aluru et al., 2017; Zhou et al., 2013). DED correlates with a loss in proteins with protective functions, such as Lf, *LCN1*, Lacrimal Proline Rich 4, lipophilin A, and lipophilin C (Jung et al., 2017; Versura et al., 2010; Zhou et al., 2009), and with an increase in serum albumin (Versura et al., 2010). Nonetheless, *LYZ* is a controversial biomarker candidate: its concentration has been reported to be either significantly decreased (Grus et al., 2005; Zhou et al., 2009) or unchanged between healthy volunteers and DED patients (Versura et al., 2010).

**Table 2:** Comparison of human tear proteome studies concerning patients affected by ocular diseases

Study	Pathology	Sample (*n*)	Collection method	Amount used	Fractioning	No. of fractions	Enzymatic digestion	Online LC column	Gradient length, flow	Spectrometer	MS mode	Data analysis software	No. of protein IDs
Winiarczyk et al. (2018)	AMD	Single subject, 1 take	STS	40 µg TP	2‐DE	ND	Trypsin	None	None	UltrafleXtreme (Bruker)	DDA	BioTools	342
Aluru et al. (2017)	DED	Pool	STS	30 μg TP	2‐DE	ND (modulated spots)	Trypsin	5 μm, 300 Å	60 min, 400 nl/min	Q‐TOF Qstar Elite (Applied Biosystems)	DDA	Protein Pilot	13
Grus et al. (2005)	DED	Single subject, 1 take	STS	20 μl	ProteinChip Array	None	None	None	None	SELDI‐TOF Ciphergen ProteinChip Reader PBS II (Ciphergen Biosystems)	Full scan	Ciphergen Express Data Manager	N/D
Huang et al. (2018)	DED	Single subject, 1 take	STS	ND	None	None	Trypsin	2.1 × 100 mm, 1.7 μm	60 min, 300 nl/min	QE Extrative (Thermo Fischer Scientific)	DDA	Proteome Discoverer	86
Jung et al. (2017)	DED	Pool	Polyester fiber rod	100 μg TP	High‐pH RP	6	Trypsin	0.75 × 500 mm, 2 μm	175 min, N/D	Q Exactive Orbitrap Hybrid (Thermo Fisher Scientific)	DDA and MRM	MaxQuant	1165
Versura et al. (2010)	DED	Single subject, 1 take	Micro‐pipette	1.5 μl	SDS‐PAGE	ND (modulated bands)	Trypsin	0.3 × 150 mm, 3 μm	45 min, N/D	Q‐TOF micro (Micromass)	DDA	Mascot	13
Zhou et al. (2009)	DED	Single subject, 1 take + pool	STS	30 μg TP	None	None	Trypsin	MudPIT:	85 min, 300 nl/min	Q‐TOF (ABI)	DDA	ProQUANT	93
								(1) 30 × 100 mm(2) 0.75 × 500 mm, 3 μm, 100 Å					
Soria et al. (2013)	DED, MGD	Pool	Polyvinyl acetate surgical sponge	300 μg TP	2‐DE	15 (modulated spots)	Trypsin	None	None	MALDI‐Ultraflex TOF/TO (Bruker)	Full scan	Mascot	15
Soria et al. (2017)	DED, MGD	Single subject, 1 take	MCT	4 μg TP	None	None	Trypsin	0.75 × 200 mm, 1.7 μm	120 min, ND	SYNAPT HDMS (Waters)	DDA	Mascot	603
Kuo et al. (2019)	DED, SS	Pool	STS	5 μg TP	None	None	Trypsin	0.75 × 150 mm	300 nl/min	HCT Ultra ETDII Ion‐trap (Bruker)	DDA	Mascot	ND
Tomosugi et al. (2005)	DED, SS	Single subject, 1 take	STS	24 μl	ProteinChip Array	None	None	None	None	SELDI‐TOF Ciphergen ProteinChip Reader PBS II (Ciphergen Biosystems)	Full scan	Biomarker Wizard	56
Csősz et al. (2012)	DR	Pool	MCT	2 µl	None	None	Trypsin	0.75 × 150 mm, 3.5 µm	200 min, 300 nl/min	4000 QTRAP (ABSciex)	DDA	ProteinPilot	53
Kim et al. (2012)	DR	Single subject, 1 take	Polyester wick	60 µg TP	2‐DE	20 (modulated spots)	Trypsin	None	None	Q‐TOF (Micromass)	DDA	Mascot	20
Pieragostino et al. (2012)	Glaucoma	Pool	STS	50 μg TP	None	None	Trypsin	0.75 × 100 mm, 3.5 μm	170 min, 250 nl/min	Q‐TOF Premier (Waters)	DDA	ProteinLynx GlobalServer	45‐15 (modulated)
		Pool	STS	150 μl	RP, 0.35 × 250 mm, 5 μm, 300 Å	33	Trypsin	0.75 × 100 mm, 3.5 μm	170 min, 250 nl/min	Q‐TOF (Waters)	DDA	Mascot	45‐15 (modulated)
Pieragostino et al. (2013)	Glaucoma		STS	80 μl	None	None	Trypsin	0.75 × 250 mm, 1.7 μm	170 min, 250 nl/min	Q‐TOF Premier (Waters)	DDA	ProteinLynx GlobalServer	27 (modulated)
Nättinen et al. (2018)	Glaucoma	Single subject, 1 take	STS	50 μg TP	None	None	Trypsin	0.75 × 100 mm, 3 μm, 120 Å	120 min, 300 nl/min	Triple TOF 5600+(ABSciex)	DDA and DIA	ProteinPilot	785
Rossi et al. (2019)	Glaucoma	Pool	STS	50 μg TP	None	None	Trypsin	0.75 × 250 mm, 5 μm	90 min, 300 nl/min	Maxis HD UHR‐TOF (Bruker)	DDA	PeptideShaker	123‐103
Acera et al. (2011)	KC	Single subject, 1 take	Micropipette	40 µg TP	2‐DE	6 (modulated spots)	Trypsin	None	None	Ultraflex TOF/TOF (Bruker)	DDA	Mascot	4
				10 µg TP	None	None	Trypsin	0.75 × 200 mm, 1.7 µm	ND	SYNAPT HDMS (Waters)	DIA	ProteinLynx GlobalServer	39
Balasubramanian et al. (2013)	KC	Pool	MCT	36–72 µl	MF10	5	Trypsin	0.75 × 100 mm, 5 μm, 200 Å	30 min, 300 nl/min	LTQ‐FT Ultra (Thermo Electron)	DDA	Mascot	75
Lema et al. (2010)	KC	Single subject, 4 takes	STS	10 µg TP	2‐DE	3	Trypsin	None	None	MALDI‐TOF MS	Full scan	Mascot	3
Pannebaker et al. (2010)	KC	Single subject, 1 take	MCT	10 µg TP	SDS‐PAGE	ND (modulated bands)	Trypsin	0.75 × 50 mm	60 min, 300 nl/min	Thermo Finnigan LTQ (Thermo Fisher Scientific)	DDA	Mascot	ND
Yenihayat et al. (2018)	KC	Pool	MCT	50 µg TP	2‐DE	9 (modulated spots)	Trypsin	None	None	MALDI‐TOF/TOF 5800 (ABSciex)	DDA	Protein Pilot	9
Kishazi et al. (2018)	TAO	Single subject, 1 take	STS	10 μg TP	OGE	12	Trypsin	0.75 × 150 mm, 5 μm, 100 Å	85 min, 220 nl/min	LTQ Orbitrap Velos (Thermo Fischer Scientific)	DDA	Proteome Discoverer	712
Jiang et al. (2020)	TAO	Pool	MCT	500 µg TP	High pH RP, 2.1 × 150 mm, 3 μm	12	Trypsin	0.75 × 400 mm, 1.9 μm	120 min, 250 nl/min	Orbitrap Fusion LUMOS (Thermo Fisher Scientific)	DDA and DIA	Spectronaut X	669

Abbreviations: 2‐DE, two‐dimensional electrophoresis; AMD, age‐related macular degeneration; DDA: data‐dependent acquisition; DED, dry eye disease; DIA, data‐independent acquisition; DR, diabetic retinopathy; FT, Fourier transform; KC, keratoconus; LTQ, linear trap quadrupole; MALDI, matrix‐assisted laser desorption ionization; MCT, microcapillary tube; MGD, meibomian gland dysfunction; ND, not defined; OGE, off‐gel electrophoresis; RP, reverse phase; SELDI, surface‐enhanced laser desorption/ionization; SS, Sjögren's syndrome; STS, Schirmer's test strip; TAO, thyroid‐associated ophthalmopathy; TOF, time‐of‐flight; TP, total proteins.

A study comparing subjects with *thyroid‐associated ophthalmopathy* (TAO), TAO + DED, DED, and controls (Matheis et al., 2015), has revealed that TAO is characterized by the upregulation of inflammatory proteins and downregulation of protective ones. It is precisely the trend of this protein panel that allows to differentiate TAO from DED. Advances in tear proteomics enabled, later on, Jiang and coworkers to deepen TAO pathogenesis by looking for potential therapeutic targets (Jiang et al., 2020). Tears collected before and after orbital decompression in patients with inactive TAO were analyzed by LC‐MS/MS, with 83 proteins resulting differentially expressed among the two disease groups and healthy controls. This study also illustrates the advantages of data‐independent acquisition (DIA). This approach bypasses precursor‐ion selection in MS/MS analyses, resulting in increased proteome coverage. Conventional data‐dependent acquisition (DDA), which uses information of the full MS scan to guide precursor‐ion selection, was first performed to generate exhaustive and sample‐specific spectral libraries. These served as a tool for subsequent DIA experiments. This approach, also called SWATH (sequential window acquisition of all theoretical mass spectra), is an attractive alternative to improve the performance of shotgun methods for label‐free, quantitative proteomics and allows for high identification rates over a larger dynamic range, enabling a more precise stratification (Ludwig et al., 2018). Then, by the combination with bioinformatics analyses, the authors were able to identify the molecular pathways more closely involved in orbital decompression, hinting to the action mechanisms underlying orbital decompression for disfiguring exophthalmos in patients with inactive TAO.

Another pathology affecting the ocular surface is *keratoconus*, an isolated disorder, which is not reported to be associated with other diseased states, and causes a progressive thinning and deformation of the cornea, affecting vision (Rabinowitz, 1998). Tears of patients affected by this ocular pathology resulted in an altered proteome profile with different expression of zinc‐α2‐glycoprotein (ZAG), Lf, and immunoglobulin kappa chain (*IGKC*) (Lema et al., 2010). The application of sodium dodecyl sulfate–polyacrylamide gel electrophoresis (SDS‐PAGE) combined with MS and proteomics analysis has confirmed and extended these results, evidencing an association between this pathology and overexpression of matrix metalloproteinase‐1 (*MMP1*), several keratins, immunoglobulins alpha and kappa, and precursors of prolactin, *LYZ*, and *LCN1* (Pannebaker et al., 2010). In general, the thinning and scarring of the cornea caused by keratoconus have been reported to be associated with decreased levels of protease inhibitors and increased levels of proteases (Acera et al., 2011; Balasubramanian et al., 2013; Lema et al., 2010; Pannebaker et al., 2010; Yenihayat et al., 2018).

Tear‐based approaches offer promising opportunities to discover biomarkers at an easily accessible source and to investigate pathologies of the ocular surface and posterior‐eye, as well as systemic diseases. Among posterior‐eye diseases, *age‐related macular degeneration* (AMD) affects the macular region in the retina, causing degenerative and neovascular changes that lead to a progressive impairment of the central vision, and represents the third leading cause of irreversible vision loss worldwide (Mitchell et al., 2018). Tear composition in AMD patients has been investigated recently, by two‐dimensional gel electrophoresis (2‐DE) combined with matrix‐assisted laser desorption/ionization (MALDI) time of flight (TOF) MS (Winiarczyk et al., 2018). This study led to the identification of 342 proteins and revealed the capabilities of tear proteomics to identify proteins related to inflammation, neovascularization, oxidative stress and impaired autophagy. Some of these proteins had been previously linked to the AMD pathology, while shootin‐1, histatin‐3, fidgetin‐like protein 1, SRC kinase signaling inhibitor, Graves’ disease carrier protein, actin cytoplasmic 1, prolactin‐inducible protein 1, and protein S100‐A7A have been implicated here for the first time and appear to be specific of the tear fluid (Winiarczyk et al., 2018).

The tear fluid also came to aid in the identification of biomarkers for *diabetic retinopathy* (DR), which may arise in patients affected by diabetes and represents a widespread microvascular complication of this pathology, with abnormal vessels formation in the retina and production of large scars (Cheung et al., 2010). In fact, it is well known that corneal wound healing and inflammation caused by vascular proliferation have a direct link with changes in tear proteome composition (Csősz et al., 2012). Tears may represent a more promising source of DR biomarkers, with respect to other biological fluids, such as blood, whose protein composition is most heavily affected by the systemic diabetic condition. MS‐based proteomics has revealed a decrease of protein concentration with disease progression and altered expression levels of several proteins, such as *LCN1*, lacritin, and Lf (Csősz et al., 2012; Kim et al., 2012). In addition, Csősz excluded proteins assignable with other clinical conditions from the candidate biomarkers of DR progression proposed, through both literature search and experimental comparisons. Of note, proteomics has also been employed to develop an efficient automated pre‐screening method that reduces time and costs of DR screening, which is currently based on digital image evaluation by human graders (Torok et al., 2013, 2015).

*Glaucoma* is correlated with an increase in intraocular pressure, leading to irreversible damage of the optic nerve. In most cases, the disease onset causes no symptoms, which is the reason why it is often called “the silent thief of sight” (Gupta & Chen, 2016). As in the case of other eye diseases, nowadays there is no cure for glaucoma, but pharmaceutical and surgical treatments that can stop the progressive vision loss. For these reasons, there is a strong need for biomarkers useful in early diagnosis and able to properly identify two distinct glaucoma sub‐types, a primary and a secondary form. Tears potentially contain helpful glaucoma biomarkers, given their location close to the trabecular meshwork, where the initial damage occurs, leading to elevated intraocular pressure (Agnifili et al., 2012). Indeed, an MS‐based proteomics approach has highlighted an upregulation of proteins involved in inflammation pathways, such as the S100 protein (Pieragostino et al., 2012). This technique has also allowed to differentiate the two glaucoma sub‐forms, the primary open‐angle (POAG) and the pseudoexfoliative glaucoma. Tear‐based proteomics may be useful in gaining new insight, not only in glaucoma pathophysiology, but also in ocular surface modifications induced by preservatives or active compounds contained in topical treatments for glaucoma management. By using SWATH and tear protein profiles from tears of individual patients, Nättinen and coworkers effectively stratified patients into less and more severe cases with good agreement with the clinical signs and symptoms (Nättinen et al., 2018).

POAG is the main form of glaucoma and represents the major cause of irreversible blindness worldwide. Considering its spreading in elderly, screening strategies and early diagnosis by tear proteomics biomarkers would be useful.

A shotgun‐proteomics approach, applied to the comparison of tears from naïve or treated POAG patients versus controls, has revealed a modulation of protein panels related to inflammation and their possible use as diagnostic and predictive biomarkers (Pieragostino et al., 2013). A pro‐inflammatory protein cargo in POAG patients has also been detected in tears and extracted extracellular vesicles (EVs) and also confirmed by targeted metabolomics analysis (Rossi et al., 2019).

### 1.4 Biomarker discovery in systemic diseases

The tear fluid is secreted directly on the ocular surface. The eye can be considered as an “appendix” of the brain and, therefore, of the central nervous system. Thus, tear proteomics has been applied for biomarker discovery, not only in ophthalmology, but also in systemic diseases, particularly those linked to neurodegeneration (described in Table 3). In particular, tears of patients affected by *multiple sclerosis* (MuS) have been analyzed by a combination of different techniques (liquid chromatography‐MS, Western Blot and ELISA), leading to the identification of only one protein with significantly increased levels in MuS, alpha‐1 antichymotrypsin (Salvisberg et al., 2014). Steps forward in this direction have been made by implementing a shotgun proteomics platform preceded by flow cytometry to sort EVs from CSF and tears (Pieragostino et al., 2019). Inflammation, angiogenesis, and immune‐response pathways were found upregulated in EVs from both CSF and tears. Similar proteomics profiles, obtained from the two examined biofluids, suggest that there is a molecular cross‐talk between CSF and tears, opening new diagnostic perspectives for this latter source of samples in MuS biomarker discovery.

**Table 3:** Comparison of human tear proteome studies concerning patients affected by systemic diseases

Study	Pathology	Sample (n)	Collection method	Amount used	Fractioning	No. of fractions	Enzymatic digestion	Online LC column	Gradient length, flow	Spectrometer	MS modes	Data analysis software	No. of protein IDs
Kalló et al. (2016)	AD	Pool	MCT	20 µg TP	SDS‐PAGE	11 bands (differentially expressed)	Trypsin	0.75 × 150 mm, 3.5 μm	90 min, 300 nl/min	4000 QTRAP (ABSciex)	DDA and SRM	ProteinPilot	19
Lebrecht et al. (2009)	Breast cancer	Single subject, 1 take	STS	60 µg TP	ProteinChip Array	20 spots (differentially expressed)	Trypsin	None	None	Q‐TOF (Micromass)	Full scan	Mascot	20
Böhm et al. (2012)	Breast cancer	Pool	STS	60 µg TP	SDS‐PAGE	32	Trypsin	None	None	MALDI‐TOF/TOF UltraflexII (Bruker)	DDA	Mascot	~150
Salvisberg et al. (2014)	MuS	Single subject, 1 take + pool	MCT	5 µg TP	OGE	ND	Trypsin	0.75 × 150 mm, 5 µm, 100 Å	85 min, 220 nl/min	LTQ Orbitrap Velos (Thermo Fisher Scientific)	DDA	EasyProt	185
Pieragostino et al. (2019)	MuS	Pool	STS	10^6^ EVs	None	None	Trypsin	0.75 × 250 mm, 5 µm	90 min, 300 nl/min	Maxis HD UHR‐TOF (Bruker)	DDA	PEAKS Studio	32–86
Boerger et al. (2019)	PD	Pool	STS	50 µg TP	SDS‐PAGE	17	Trypsin	0.5 × 150 mm	50 min	LTQ Orbitrap XL (Thermo Fisher Scientific)	DDA	MaxQuant	571

Abbreviations: AD, Alzheimer's disease; DDA, data‐dependent acquisition; EV, extracellular vesicles; LTQ, linear trap quadrupole; MALDI, matrix‐assisted laser desorption ionization; MCT, microcapillary tube; MuS, multiple sclerosis; ND, not defined; OGE, off‐gel electrophoresis; PD, Parkinson's disease; SRM, single reaction monitoring; STS, Schirmer's test strip; TOF, time‐of‐flight; TP, total proteins.

Recently, tear proteomics has been focusing also on *Alzheimer's disease* (AD) (Kalló et al., 2016) and *Parkinson's disease* (PD) (Boerger et al., 2019; Maass et al., 2020). These neurodegenerative disorders can also be counted as systemic pathologies because their symptoms extend beyond the central nervous system, resulting in systemic abnormalities (Choong et al., 2015; Wang et al., 2017). In particular, a tetra‐panel of four tear proteins (*LCN1*, dermcidin, *LYZ*, and lacritin) has been reported to be specific to AD diagnosis, with an 81% sensitivity and 77% specificity (Kalló et al., 2016). A targeted workflow by selected reaction monitoring (SRM) on a triple‐quadrupole mass analyzer was employed in that study. Due to their accuracy, precision, and versatility, SRM and multiple reaction monitoring (MRM) are still considered the gold standards for MS‐based quantitation, allowing for simultaneous monitoring of biomarker panels from a single sample (Kontostathi et al., 2019; Meyer & Schilling, 2017).

Differently from AD, PD is characterized by the accumulation of Lewy bodies not only in the brain, but also throughout the central and peripheral nervous systems, as well as in other organs (Iacono et al., 2015). Comparing the basal tears of PD patients and healthy controls, 21 proteins were reported to be significantly upregulated, whereas 19 were significantly downregulated. These alterations involve proteins of the immune response, lipid metabolism, and oxidative‐stress pathways (Boerger et al., 2019).

Tear proteome profiling by MS‐based proteomics has also been reported to allow the discrimination between healthy controls and age‐matched women affected by *breast cancer*, with a specificity and sensitivity of approximately 70% (Lebrecht et al., 2009). A subsequent study highlights the involvement of proteins of the immune‐response pathways (e.g., C1Q1 and S100A8) and of some metabolic cascades (e.g., ALDH3 and TPI), depicting affected networks in breast cancer patients versus controls (Böhm et al., 2012).

## 2 POOLED VERSUS SINGLE‐TEAR ANALYSIS

To achieve a wide coverage, tear proteomics analysis has to face the difficulties related to the relatively small volume of sample that can be collected. In three out of five studies on healthy human tears, samples from different volunteers were pooled together to increase the sample volume to handle and the proteome coverage (Aass et al., 2015; Dor et al., 2019; Zhou et al., 2012). In these studies, sample pooling is based on the assumption that the main composition of body fluids is likely to have strong similarities among healthy donors, as it has been shown also in the case of urine (Adachi et al., 2006). Pooling has the advantages of increasing the sample quantity available for the analysis, reducing inter‐ or intra‐subject background noise, and decreasing the number of runs to be performed. Nonetheless, sample identity and potentially meaningful individual variability are unavoidably lost by pooling. An alternative approach consists in the pooling of sequential tear collections from a single subject, as performed by de Souza et al. (2006). Nevertheless, this approach loses information on the intra‐ and inter‐day variability of the tear proteome and implies an averaging over time. Furthermore, the powerful implementation of predictive and diagnostic biomarkers requires quantitative, time‐resolved information on individual protein profiles.

In spite of the small sample volume that can be collected from each eye (between 3 and 10 μl) (Palakuru et al., 2007; Rentka et al., 2017; Scherz et al., 1974), the recent technological improvements (nano‐chromatography coupled to high‐sensitivity, high‐resolution, and high‐speed mass spectrometers) has allowed to increase the number of identified proteins in a single sample, from about 50 proteins (Li et al., 2005) to approximately a thousand (R. Grandori et al., personal communication, 2021), making single‐tear analysis an actual and attractive frontier of clinical proteomics.

This approach would be particularly relevant for case studies, but has a potential also for population‐based studies. The subject‐to‐subject variability highlights the importance of characterizing not only disease versus health, but also the individual profiles, in line with the ambition and always more widespread practice of personalized medicine (Gerner et al., 2020; Goetz & Schork, 2018; Hagan et al., 2016; Kowalczyk et al., 2020; Macklin et al., 2020). The high resolution and sensitivity of state‐of‐the‐art MS instrumentations have improved the precision of proteomics profiles (Flores‐Morales & Iglesias‐Gato, 2017) and can dispense with pooled samples, the mandatory condition for an actually personalized medicine. In this context, the single‐tear analysis is standing out as an interesting, so‐called “nonconventional sample,” applicable to a single subject for obtaining personalized profiles (Gerner et al., 2020; Licier et al., 2016).

It is also worth mentioning that the individual, single‐tear approach would also allow to investigate the dynamics of the tear proteome, which is expected to undergo significant variations according to external and internal factors beyond the physiopathological state, such as the use of drugs or specific eye drops (Ji et al., 2019; Karnati et al., 2013; Nättinen et al., 2018), dieting (Jalbert, 2013), day time (Ng et al., 2000), and type of tear collected (reflex, open‐eye, closed‐eye, as discussed previously) (Sitaramamma et al., 1998). Single‐tear analysis is also required to establish standard collection conditions to yield highly controlled, comparable data. Despite these important observations, the work by Li and coworkers is the only example of tear proteome investigation employing single‐tear MS‐based analysis reported in the literature (Li et al., 2005). Thus, the development of proteomics approaches capable of yielding a deep tear‐fluid characterization on single withdrawals from single subjects and employing very low sample volumes is an urgent demand in this field. The following sections discuss the main steps of the current procedures and the most critical technical aspects.

## 3 TEAR COLLECTION METHODS

The method employed for sample collection has an impact on tear composition (Rentka et al., 2017), in particular on proteins (Green‐Church et al., 2008; Nättinen et al., 2020). These studies suggest a careful evaluation of the tear‐collection methods, according to the experimental design and to the focus of the analysis. The most common sampling methods are those by Schirmer's test strips (STSs) or microcapillary tubes (MCTs). To date, most of the proteomics investigations employed STSs (Aass et al., 2015; Dor et al., 2019; Zhou et al., 2012). MCTs is described only in two studies (de Souza et al., 2006; Li et al., 2005). A possible reason is that, despite MCT‐based sampling being less invasive, safer, and not inducing reflex tearing, it requires a trained specialist (as described in Table 4). A recent MS‐based study highlights differences in the protein profiles between MCT‐ and STS‐collected samples (Nättinen et al., 2020).

**Table 4:** Pros and cons of the two most common sample collection methods

	Schirmer's strip	Microcapillary tubes
Pros	Easier to handle	Less invasive
		Safe
		No reflex tearing
	More pleasant for the volunteers	
Cons	Reflex tearing	Require a trained specialist
	Binding and retention depend on MW and hydrophobic surface area of proteins	
		Sampling is interrupted by blinking
	Can injure the conjunctival surface and microvasculature	

The different collection methods led to similar counts of quantified proteins and to a good protein identification overlap. Despite these similarities, MCT samples led to the identification of more extracellular proteins (immune response pathway), while STS samples contained more intracellular proteins (e.g., heat‐shock proteins, annexins, and S100 proteins).

### 3.1 STS collection

STSs are strips of filter paper that are placed in the conjunctival sac, to absorb the tear fluid. Being in direct contact with the conjunctiva, it can cause irritation and undesired lacrimation (Choy et al., 2001). Reflexive tearing affects the protein concentrations of the collected samples, which are diluted in an uncontrolled way (Sack et al., 2003). Subsequent sample handling and protein extraction are challenging and can further increase the variability of the results (Feist & Hummon, 2015). In fact, these steps involve strip cutting and repeated sample transferring, with the risk of protein loss and contamination. All the authors employing STSs avoided the use of local anesthesia, because it decreases tear production (Nwaji & Barrah, 2011). Another critical aspect is the sample preparation steps after tear collection. Several extraction conditions were proposed, by varying extraction solvent, volume, time, and temperature of STS incubation (Aass et al., 2015; Zhou et al., 2012). Dor et al. (2019) employed a different extraction method, based on strip centrifugation. In this case, the centrifugal force pulls the tear fluid out of the strip bypassing buffer addition (Kishazi et al., 2018; Posa et al., 2013). Despite the variety of extraction strategies described in the literature, it has been reported that these steps can bias both protein identification and quantitation, due to noncovalent protein–strip interactions that cannot be overcome by diffusion or centrifugation (Denisin et al., 2012).

Nonetheless, the application of STS collection method is straightforward, and does not require any trained specialist to be performed. Moreover, subjects experiencing STS and MCT collection methods reported that STS collection is less unpleasant than MCT, likely due to the flexibility of STSs compared with the rigidity of MCTs (Rentka et al., 2017). To facilitate STS transport and storage on a large scale and for long periods of time, Qin and coworkers developed a drying method for strips that could increase tear availability for research scopus and prepare the ground for tear biobanks (Qin et al., 2017).

### 3.2 MCT collection

MCTs are hollow cylinders made of glass or plastic. The MCT is placed horizontally to the side of the conjunctival sac, so that the tear fluid can flow through by capillarity. MCT sampling does not require any extraction procedure, but it must be performed by a trained specialist. Typically, this method allows the collection of a 3–5 µl of tear, a volume which is not so different from the total volume (7–10 µl) typically present on the human eye (Rentka et al., 2017). The collection typically takes a few minutes, a time that roughly corresponds to the time necessary for the eye to produce a few microliters of tears under normal conditions, the secretion velocity being 0.5–2.2 µl/min (Rentka et al., 2017). The two published studies based on MCT collection have been carried out on either open (Li et al., 2005) or closed eye (de Souza et al., 2006). Nevertheless, MCT sampling guarantees the collection of nonstimulated tears only, which yield the most reliable quantitation of tear composition (Rentka et al., 2017).

## 4 SAMPLE FRACTIONATION

Many authors choose to reduce sample complexity by upstream analytical strategies. Sample fractionation before online separation by liquid chromatography (LC) is a common procedure adopted in proteomics, allowing for highest signal‐to‐noise ratios, possibly at the expense of protein losses. This approach improves chances to detect low abundance proteins and also provides additional information on proteome components, such as molecular weight, hydrophobicity, or isoelectric point (Ball & Roulhac, 2010; Mostovenko et al., 2013). Nonetheless, it should be considered that additional steps in the protocol may represent another source of experimental error and impact on the analysis costs. In particular, it might be undesirable for single‐tear analysis due to the small sample amounts. In this case, a greater benefit could derive, instead, by the ultrahigh resolution in MS analysis, high performance and automation in LC separation, and flexibility in scan modes (DIA and DDA) made possible by the recent technological advances.

For tear analysis of healthy human subjects three fractionation procedures have been employed so far: protein separation by SDS‐PAGE (de Souza et al., 2006; Li et al., 2005), peptide separation by offline strong cation exchange chromatography (SCX) (Aass et al., 2015; Zhou et al., 2012) and off‐gel electrophoresis (OGE) (Dor et al., 2019).

### 4.1 SDS‐PAGE separation

Electrophoresis can be combined with MS‐based proteomics studies by different approaches. Li performed SDS‐PAGE on 1 µl of tear fluid, followed by in‐gel digestion of the bands evidenced by silver staining (10 bands) and subsequent MALDI‐MS/MS sequencing (Li et al., 2005). De Souza applied the so‐called GeLC‐MS approach (Makridakis & Vlahou, 2018), consisting of 1D gel separation combined with MS analysis (de Souza et al., 2006). Two gel lines, each loaded with 4 µl of tears and cut in 13 sections, were processed by in‐gel digestion and peptides extraction before nanoLC‐MS analysis. Gel‐based approaches are simple to perform and can help tackle sample complexity, especially when 2D electrophoresis is employed. However, these approaches have some intrinsic drawbacks. In fact, gel separation of very high‐ or low‐molecular‐weight proteins can be particularly inefficient, as well as extreme pIs on 2D gels. Moreover, in‐gel digestion introduces additional sources of variability because it is more complex and time‐consuming than in‐solution digestion. Furthermore, gel electrophoresis introduces a bottleneck in the dynamic range of the overall analytical procedure. Due to these limitations, recent studies have adopted gel‐free approaches for high‐throughput analysis.

### 4.2 Offline SCX separation

In the examples of the previous paragraph, the upstream fractionation procedure is performed at the protein level. On the contrary, offline SCX is applied at the peptide level, following in‐solution digestion of raw samples (Aass et al., 2015; Zhou et al., 2012). Elution is achieved by salt (Aass et al., 2015) or pH gradient (Zhou et al., 2012). The offline SCX fractionation is the fastest alternative among the three reported methods for a pre‐fractionation in tear fluid analysis, but its effectiveness in proteome coverage improvement is controversial: SCX seems to offer the highest proteome coverage when compared with IEF and GeLC (Mostovenko et al., 2013), although it is less efficient than reverse phase chromatography at high pH (Manadas et al., 2009).

### 4.3 OGE separation

Both SCX and OGE separation are usually performed at the peptide level and exploit the peptide pIs, but they lead to different subsets of identified proteins (Antberg et al., 2012). OGE is yet scarcely applied to human tear proteomics and it has been employed only by one research team (Dor et al., 2019). Compared with SCX, OGE has led to lower numbers of protein identifications and to lower run‐to‐run reproducibility (Antberg et al., 2012).

**Figure 1:**
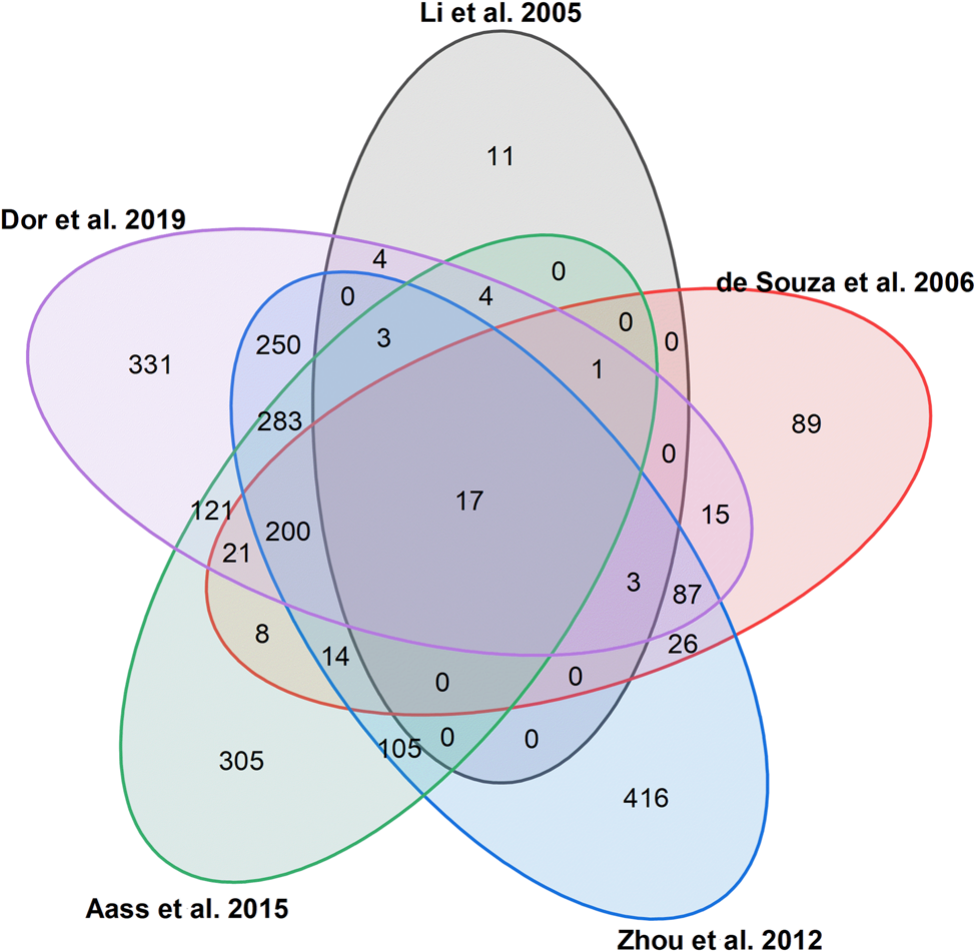
Venn diagram of the five human tear proteome studies concerning healthy subjects published since 2005. The five protein lists were converted into gene names by using the UniProtKB Retrieve/ID mapping tool and compared [Color figure can be viewed at wileyonlinelibrary.com]

Each separation method provides complementary information and leads to the identification of different numbers and subsets of tear proteins (see Figure 1). This point highlights the advantage of merging different approaches to improve deepness of tear proteomics.

## 5 DIGESTION AND LC‐MS/MS ANALYSIS

Most studies make use of trypsin for protein digestion. However, the combination of different enzymes has proven its efficiency in increasing the identification rate (de Souza et al., 2006; Saveliev et al., 2013). We adduce as examples two recent studies (Aass et al., 2015; Dor et al., 2019), which employ the same mass spectrometer (LTQ‐Orbitrap) and only differ for the digestion protocol. Combining Lys‐C and trypsin for enzymatic digestion has allowed Aass to enhance the number of identified proteins from 1351 to 1526 (Aass et al., 2015; Dor et al., 2019).

Even the optimization of online peptide separation could improve tear proteome coverage (Shishkova et al., 2016). Many aspects of the chromatographic separation are subjected to possible variations, such as column length, particle size, and gradient shape (described in Table 1). However, due to the different instrumental configuration and parameters adopted, it is difficult to draw conclusions about the most effective LC setting among the examined studies. Multidimensionality is another aspect that has not yet been systematically explored and may bring further benefits to LC platforms for tear samples (Ferrari et al., 2017). The combination of two or more orthologous LC steps increases column peak capacity and selectivity and, thus, the resolving power and identification performance of the analytical procedure.

The advent of Orbitrap mass analyzers in 2005 has carried around a powerful innovation in the technological panorama for omics sciences, offering exceptional resolution, robustness, versatility, sensitivity, and accuracy of MS analyses. The Orbitrap technology is still rapidly evolving (Hecht et al., 2019). Nevertheless, these advantages have not yet led to improvements in the number of identifications in tear analysis over the last 8 years (Aass et al., 2015; Dor et al., 2019; Zhou et al., 2012). There is a fairly good overlap among these studies (see Figure 1), despite the variability induced by sample collection, fractionation, enzymatic digestion (Walmsley et al., 2013), and LC‐MS methods (Tabb et al., 2010). However, these results indicate that protocol refinement is still needed for high‐performance tear proteomics.

## 6 CONCLUSIONS

This review summarizes the most relevant differences in MS‐based approaches to tear proteome characterization. By merging all the protein lists published so far, it is possible to compile high‐confidence identification (FDR ≤ 1% and at least two unique peptides for each protein, excluding keratins and non‐reviewed proteins) of 1620 proteins in human tear fluid (Dor et al., 2019).

The field is benefiting from rapid technological advances and has moved progressively from gel‐based to gel‐free strategies. Despite the major efforts and technical advances, the large dynamic range still represents a challenge in the search for biomarkers by MS‐based tear proteomics. Transferring the results of biomarker discovery into clinical practice is not trivial (Subramanyam & Goyal, 2016) and such difficulties slow down progress also in the case of tear fluid analysis. To overcome this limitation, the current trend is to focus on protein panels rather than on single proteins. The implementation of high‐performance, gel‐free and label‐free, quantitative proteomics of the tear fluid will certainly open new avenues in this direction.

Another issue is the paucity of studies on in‐depth analysis of single‐tear samples, which are hindered by the limited sample amounts. However, diagnostics and treatment in modern medicine have been progressively focusing on disease subtyping and patient stratification, based on better‐defined and better‐integrated omics profiles (Vlahou, 2019). Such a level of molecular profiling can guide personalized evaluation within a given physio‐pathological state. Furthermore, insights on the underlying molecular, pathogenic mechanisms offer the possibility to define individual disease endotype, that is, the specific link between genotype and phenotype (Agache & Akdis, 2019). Nowadays, precision, and personalized medicine is limited mainly to genetic approaches (Olivier et al., 2019). This field still awaits effective integration with individual profiling at the transcriptome and proteome levels. Thanks to the recent methodological advances, MS‐based tear proteomics can guarantee the high accuracy and sensitivity necessary for biomarker discovery and single‐tear analysis. Thus, tear proteomics is expected to play in the near future a strategic role, not only by the identification of innovative, low‐invasiveness biomarkers, but also by feeding the three pillars of personalized medicine: accurate molecular profiles, noninvasive samples, and endotype characterization.

## Supporting information

Supporting information.
