# Supplementary material for: Mass spectrometry‐based tear proteomics for noninvasive biomarker discovery
Source: Mass Spectrom Rev. 2021 Mar 24;41(5):842–60. doi: 10.1002/mas.21691 (PMC9543345; doi:10.1002/mas.21691)
Supplement: Supplementary file 1 — Supporting information. [file MAS-41-842-s001.docx]

**Table S1.** Comparison of human tear proteome studies concerning patients affected by ocular diseases. Only the proteins indicated by the authors as candidate or validated (e.g. ELISA) biomarkers are reported.

| Disease | Study | Biomarker candidates |
| --- | --- | --- |
| AMD | (Winiarczyk et al., 2018) | Actin cytoplasmic 1  Fidgetin-like protein 1  Graves disease carrier protein  Histatin-3  Prolactin-inducible protein 1  Protein S100-A7A Shootin-1 SRC kinase signaling inhibitor |
| DED | (Aluru et al., 2017) | Cystatin SN precursor  Ecto-ADP ribosyltranferase -5-precursor  Heterogeneous nuclear ribonucleoprotein Q isoform 6  Keratin type II cytoskeletal protein  Lactotransferrin isoform 1 precursor  Rho-related GTP-binding protein RhoQ precursor/RhoJ precursor  Ribonuclease p protein subunit 20 and protocadherin  SHC transforming 1 isoform |
|  | (Grus et al., 2005) | Alpha-1-Antitrypsin, C-terminal fragment  Nasopharyngeal carcinoma-associated proline-rich protein  Proline rich protein 3  Proline-rich protein 4  S100 A8 (calgranulin A)  Unidentified 3700 Da protein  Unidentified 3916 Da protein |
|  | (Huang, Du, & Pan, 2018) | Annexin A1  Complement component 3  Deleted in malignant brain tumors 1  Heat shock 27kDa protein 1  Immunoglobulin J polypeptide  Keratin 1  Lacritin  Lactotransferrin  Lipocalin 1  Lysozyme  Polymeric immunoglobulin receptor  Proline rich 4 (lacrimal)  S100 calcium binding protein A8  S100 calcium binding protein A9  Secretoglobin, family 2A, member 1  Transferrin  Zinc-alpha-2-glycoprotein |
|  | (Jung et al., 2017) | Lactotransferrin  Lipocalin 1  Lysozyme |
|  | (Versura et al., 2010) | Lactoferrin  Lipocalin 1  Lipophilin A-C  Serum albumin |
|  | (Lei Zhou et al., 2009) | Alpha-1-acid glycoprotein 1  Alpha-enolase  Lactoferrin  Lipocalin 1  Lysozyme  Prolactin-inducible protein  S100 A11 (calgizzarin)  S100 A4  S100 A8 (calgranulin A)  S100 A9 (calgranulin B) |
|  | (Soria et al., 2013) | Annexin A1  Annexin A11  Cystatin-S  Phospholipase A2-activating protein  S100 A6 |
|  | (Soria et al., 2017) | Alpha-1-antitrypsin  Antileukoproteinase  Ig gamma-1  Lactoperoxidase  Membrane-associated phospholipase A2  Thioredoxin |
|  | (Kuo et al., 2019) | Lacritin  Lactoferrin  Lipocalin 1  MMP-9  Prolactin-inducible protein |
|  | (Tomosugi et al., 2005) | 2094 m/z  2743 m/z  3483 m/z  4972 m/z  10860 m/z  14191 m/z  14702 m/z  16429 m/z  17453 m/z  17792 m/z |
| MGD | (Soria et al., 2013) | Annexin A1  Annexin A11  Cystatin-S  Phospholipase A2-activating protein  S100 A6 |
|  | (Soria et al., 2017) | Alpha-1-antitrypsin  Antileukoproteinase  Ig gamma-1  Lactoperoxidase  Membrane-associated phospholipase A2  Thioredoxin |
| DR | (Csősz et al., 2012) | Immunoglobulin lambda chain  Lacritin  Lactotransferrin  Lipocalin 1  Lipophilin A  Lysozyme C |
|  | (Kim et al., 2012) | Beta-2 microglobulin  Heat shock protein 27  Lipocalin 1 |
| Glaucoma | (Pieragostino et al., 2012) | Immunoglobulins  Lipocalin 1  Lysozyme C  Prolactin Inducible Protein  Protein S100 |
|  | (Pieragostino et al., 2013) | Actin, cytoplasmic 1  Actin, cytoplasmic 2  Beta-2-microglobulin  Cystatin-S  Heat shock protein beta-1  Ig alpha-1 chain C region  Ig alpha-2 chain C region  Ig gamma-3 chain C region  Ig kappa chain C region  Immunoglobulin J chain  Keratin, type II cytoskeletal 1  Lactotransferrin  Lipocalin 1  Lysozyme C  Peroxiredoxin-1  Polymeric immunoglobulin receptor  POTE ankyrin domain family member  POTE ankyrin domain family member  POTE ankyrin domain family member E  POTE ankyrin domain family member F  Prolactin-inducible protein  Proline-rich protein 1  Proline-rich protein 4  Serotransferrin  Serum albumin  Zinc-alpha-2-glycoprotein  Zymogen granule protein 16 homolog B |
|  | (Nättinen et al., 2018) | 14-3-3 protein zeta/delta  14-3-3 protein epsilon  Ubiquitin carboxyl-terminal hydrolase isozyme L3  Profilin-1  Platelet-activating factor acetylhydrolase IB subunit alpha2  Acyl-CoA-binding protein |
|  | (Rossi et al., 2019) | Lysozyme C  Polymeric immunoglobulin receptor  Antileukoproteinase  Thioredoxin |
| KC | (Acera et al., 2011) | Cystatin-S  Ig-κ chain C region  Ig J chain  Lipocalin-1 |
|  | (Balasubramanian et al., 2013) | Cathepsin B  Polymeric immunoglobulin receptor  α-Fibrinogen  Cystatin SN  Cystatin S  Keratin, type I cytoskeletal 14  Keratin, type II cytoskeletal 5 |
|  | (Lema et al., 2010) | Zinc-α2-glycoprotein  Lactoferrin  Immunoglobulin kappa chain |
|  | (Pannebaker, Chandler, & Nichols, 2010) | Matrix metalloproteinase 1  Tissue inhibitor of metalloproteinase 1  Tumor necrosis-related apoptosis-inducing ligand-R1  Immunoglobulin kappa chain  Immunoglobulin alpha chain  Prolactin precursor  Lysozyme C  Lipocalin |
|  | (Yenihayat et al., 2018)* | Keratin, type II cytoskeletal 1  Alpha-1 antitrypsin  Serum albumin  Ig gamma-1 chain C region  Lysozyme C  Glyceraldehyde-3-phosphate dehydrogenase  Keratin, type I cytoskeletal 10  Apolipoprotein A-I  Lipocalin-1 |
| TAO | (Kishazi et al., 2018)* | Alpha-1-antichymotrypsin  Cystatin-C  NAD(P)H dehydrogenase  Haptoglobin  Thioredoxin domain-containing protein 5  Phospholipase A2, membrane associated  Signal transducer and activator of transcription 1-alpha  Protein ABHD14B  Retinal dehydrogenase 1  Alcohol dehydrogenase class-3 |
|  | (Jiang et al., 2020)* | Inter-alpha-trypsin inhibitor heavy chain H3  Ribonuclease T2  Unconventional myosin-VI  Immunoglobulin lambda-like polypeptide 5  Calpastatin  Immunoglobulin heavy variable 1-3  Lamin B1, isoform CRA_a  Polypyrimidine tract-binding protein 1  Selenoprotein P  Coatomer subunit delta |

*considering the high number of candidate biomarkers proposed, only the 10 proteins with the highest differential expression are reported

**Table S2.** Comparison of human tear proteome studies concerning patients affected by systemic diseases. Only the proteins indicated by the authors as candidate or validated (e.g. ELISA) biomarkers are reported.

| Disease | Study | Biomarker candidates |
| --- | --- | --- |
| AD | (Kalló et al., 2016) | Prolactin-inducible protein  Lysozyme-C  Lactotransferrin  Lipocalin-1  Extracellular glycoprotein lacritin  Dermcidin |
| Breast cancer | (Lebrecht et al., 2009) | *20 unidentified biomarkers (SELDI-TOF-MS on tear samples)* |
|  | (Böhm et al., 2012)* | Extracellular sulfatase Sulf-1  Cystatin-SA  5-AMP-activated protein kinase subunit γ-3  Triosephosphate isomerase  Microtubule-associated tumor suppressor 1  Transferrin receptor protein 1  Keratin, type I cytoskeletal 9  Putative lipocalin 1-like protein 1  Malate dehydrogenase, cytoplasmic  GTP-binding protein Di-Ras2 |
| MuS | (Salvisberg et al., 2014) | Alpha-1 antichymotrypsin  Zymogen granule protein 16 homolog B  Proline-rich-protein 4 |
|  | (Pieragostino et al., 2019)* | Transforming growth factor beta 1  N-myc proto-oncogene protein  Interleukin 5  Serum response factor  Nuclear factor erythroid 2-related factor 2  Hypoxia-inducible factor 1-alpha  Epidermal growth factor receptor  Cyclic ADP ribose hydrolase  Angiopoietin-2  Myc |
| PD | (Boerger et al., 2019)* | Keratin, type I cytoskeletal  GDP-mannose 4,6 dehydratase  Actin-related protein 2  Lactoperoxidase  Heat shock protein HSP 90-alpha  Basement membrane-specific heparan sulfate proteoglycan core protein  Zymogen granule protein 16 homolog B  Myosin-14  Extracellular glycoprotein lacritin  Ig alpha-2 chain C region |

*considering the high number of candidate biomarkers proposed, only the 10 proteins with the highest differential expression are reported
